# Sappanwood extract modulates hepatic structure–function in hepatomegaly and hepcidin related iron regulatory pathways in a phenylhydrazine induced hemolytic anemia rat model relevant to thalassemia

**DOI:** 10.1002/ame2.70264

**Published:** 2026-08-05

**Authors:** Mohammad Indra Pratama, Ratu Safitri, Desak Made Malini, Mas Rizky A. A. Syamsunarno, Mohammad Ghozali, Madihah Madihah

**Affiliations:** ^1^ Department of Biology, Faculty of Mathematics and Natural Sciences Universitas Padjadjaran Jatinangor Indonesia; ^2^ Department of Biomedical Sciences, Faculty of Medicine Universitas Padjadjaran Jatinangor Indonesia

**Keywords:** *Caesalpinia sappan* L., hemolytic anemia, hepatomegaly, hepcidin, phenylhydrazine, thalassemia

## Abstract

**Background:**

Hepatomegaly in hemolytic disorders, including in thalassemia, is a common complication, primarily due to hemosiderosis and activation of extramedullary hematopoiesis. Globin chain imbalance leads to chronic hemolysis and ineffective erythropoiesis, which are associated with suppression of hepcidin expression. Reduced hepcidin levels may increase iron absorption and accumulation, contributing to oxidative stress and liver tissue damage. Iron chelation therapy, such as deferipron, is effective but has side effects, so safer alternatives are needed. Sappanwood (*Caesalpinia sappan* L.) contains the active compound brazilin, which chelates iron and exhibits antioxidant activity, potentially reducing oxidative stress, improving iron metabolism disorders, and reducing hepatomegaly. This study aims to evaluate the potential of sappanwood extract as a natural iron chelator and antioxidant.

**Methods:**

This experimental study used male Wistar rats (180–250 g) with a model of iron overload hemolytic anemia, which were divided into normal controls, negative controls (phenylhydrazine 40 mg/kg BW), positive controls (deferiprone 75 mg/kg BW), and sappanwood extract (SWE) treatment (50, 100, 200 mg/kg BW) for 14 days. The parameters measured included iron metabolism, hepatomegaly, liver function, hepcidin‐related gene expression, and oxidative stress. Data were analyzed using ANOVA, with *p* < 0.05 considered significant.

**Results:**

The results showed that administration of 100 mg/kg BW SWE provided optimal improvement (*p* < 0.05), including reductions in hepatomegaly, SGPT, hemosiderin, lipid peroxidation, bilirubin, extramedullary hematopoiesis, and modulation in iron‐related gene expression, including *Hamp, Bmp6, Epo*, and *Ftl*.

**Conclusions:**

These findings confirm the potential of sappanwood as a potential therapeutic candidate for the management of iron overload in thalassemia.

## Graphical abstract

Phenylhydrazine (PHZ) induces hemolytic anemia characterized by increased erythrocyte destruction and ineffective erythropoiesis, leading to hepcidin suppression through disruption of the BMP/SMAD signaling pathway. Reduced hepcidin levels enhance intestinal iron absorption and hepatic iron accumulation, leading to hepatic iron overload. Excess iron promotes reactive oxygen species (ROS) generation and lipid peroxidation, triggering oxidative stress, elevated SGPT and bilirubin levels, and activation of extramedullary hematopoiesis, ultimately contributing to hepatomegaly and impaired liver structure–function. Administration of sappanwood extract (SWE), rich in the bioactive compound brazilin, exerts iron‐chelating and antioxidant activities. SWE modulates hepcidin‐related gene expression (*Hamp, Bmp6, Epo, and Ftl*), reduces hepatic iron deposition and oxidative stress (↓ MDA), decreases SGPT and bilirubin levels, suppresses extramedullary hematopoiesis, and ameliorates hepatomegaly, thereby restoring hepatic structure–function in PHZ‐induced hemolytic anemia rats.

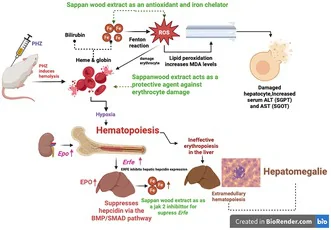

## 1 INTRODUCTION

Hepatomegaly is a condition in which the liver becomes enlarged. Several factors can cause this enlargement. These include metabolic disorders, blood flow issues, cancer cell growth, infections, toxin exposure, and inflammation.^1^ Hepatomegaly is one of the most common findings in patients with thalassemia. In a study of 104 patients with thalassemia, 46% had hepatomegaly. Another study found that among 158 patients with thalassemia, 62.65% had hepatomegaly. The prevalence of hepatomegaly also increased along with serum ferritin levels. Among patients with serum ferritin below 1500 ng/mL, the prevalence was 53.94%. In those with levels above 1500 ng/mL, the prevalence increased to 70.73%.^2^, ^3^ In thalassemia, abnormal hemoglobin causes young red blood cells (erythroblasts) to break down and die before maturing into adult red blood cells, resulting in hemolytic anemia and ineffective erythropoiesis, which triggers iron accumulation in the liver.^4^ Excess iron from red blood cell destruction and transfusions in thalassemia can cause inflammation and oxidative stress, which can harm liver cells. The iron molecules participate in the Fenton reaction, leading to the accumulation of reactive oxygen species (ROS) and liver cell injury. This results in an enlarged liver, known as hepatomegaly.^3^, ^5^, ^6^

In addition to iron accumulation in the liver, hemolytic anemia and ineffective erythropoiesis also stimulate extramedullary hematopoiesis (EMH).^7^, ^8^ Hemolytic anemia in thalassemia promotes increased production of erythroid progenitor cells, which leads to increased intestinal iron absorption and ultimately storage in various organs of the body to help form more erythrocytes.^9^ Chronic anemia in patients with thalassemia also reduces hepcidin levels, thereby impairing erythropoiesis. Increased hematopoietic activity due to ineffective erythropoiesis often leads to extramedullary hematopoiesis, which can cause hepatomegaly.^5^, ^10^ In conditions of hypoxia and hemolytic anemia, there is an increase in erythroferrone (ERFE) protein in response to erythropoietin (*Epo*). This protein inhibits hepcidin expression by interacting with the BMP‐SMAD signaling pathway in the liver. Hepcidin levels are influenced by bone morphogenetic proteins (BMPs), especially BMP6, as well as SMAD (Small Mothers Against Decapentaplegic) proteins, which play an essential role in maintaining iron homeostasis by regulating *Hamp* (hepcidin) gene transcription.^11^

Erythropoiesis requires an adequate supply of iron, which is regulated by the hormone hepcidin. Erythroferrone (ERFE), secreted by erythroblasts in response to increased erythropoietin, suppresses hepcidin production to increase iron availability. In thalassemia, globin chain imbalance causes severe ineffective erythropoiesis and excessive ERFE production, thereby excessively suppressing hepcidin and triggering progressive systemic iron accumulation.^12^ In cases of iron overload, deferiprone or deferoxamine will be administered as chelators to prevent poisoning and adverse effects. However,^13^ side effects after administering deferiprone at 20 mg/kg BW and deferoxamine at 75 mg/kg BW per day have been reported in children, including increases in urea and creatinine, indicating kidney dysfunction. Serious side effects reported include agranulocytosis and neutropenia. Long‐term use has also been associated with a variety of side effects, including arthralgia, nausea, vomiting, headache, and visual disturbances.^14^, ^15^ Ruxolitinib is used to suppress ineffective erythropoiesis by inhibiting JAK2. However, its use (10 mg/kg twice daily) carries the risk of lowering immunity, increasing the risk of herpes zoster reactivation, non‐melanoma skin cancer and neurological disorders.^16^, ^17^ Therefore, research on iron chelators with minimal side effects is needed. Sappanwood is a plant belonging to the Caesalpiniaceae family commonly found in Indonesia, where it is often consumed as a health drink.^18^ Brazilin is the primary compound in sappanwood with iron‐chelating and antioxidant properties. Sappan also contains phytochemicals, including the homoisoflavonoid brazilin, sappanone A, protosappanin, and hematoxylin, which have antioxidant and anti‐inflammatory properties and the potential to protect the liver. In addition, brazilin may be a potential inhibitor of JAK2 in the JAK2/STAT5 pathway.^19^ It also forms hexadentate complexes with iron in a 1:1 ratio, thereby preventing the formation of free radicals. These properties makes sappan a potential alternative iron chelating agent, given its minimal side effects. Several studies have reported that sappanwood extract (SWE) can prevent, reduce, and repair liver damage.^20^, ^21^ Research on the therapeutic potential of sappanwood, particularly its active compound, brazilin, for treating hepatomegaly in hemolytic anemia remains limited. The molecular mechanisms by which brazilin mitigates oxidative stress and extramedullary hematopoiesis also remain unclear. Therefore, this study aims to evaluate the efficacy of brazilin‐rich sappan extract and elucidate its mechanisms as a potential hepatoprotective and iron‐modulating therapy.

## 2 METHODS

### 2.1 Chemicals and reagents

**FIGURE 1:**
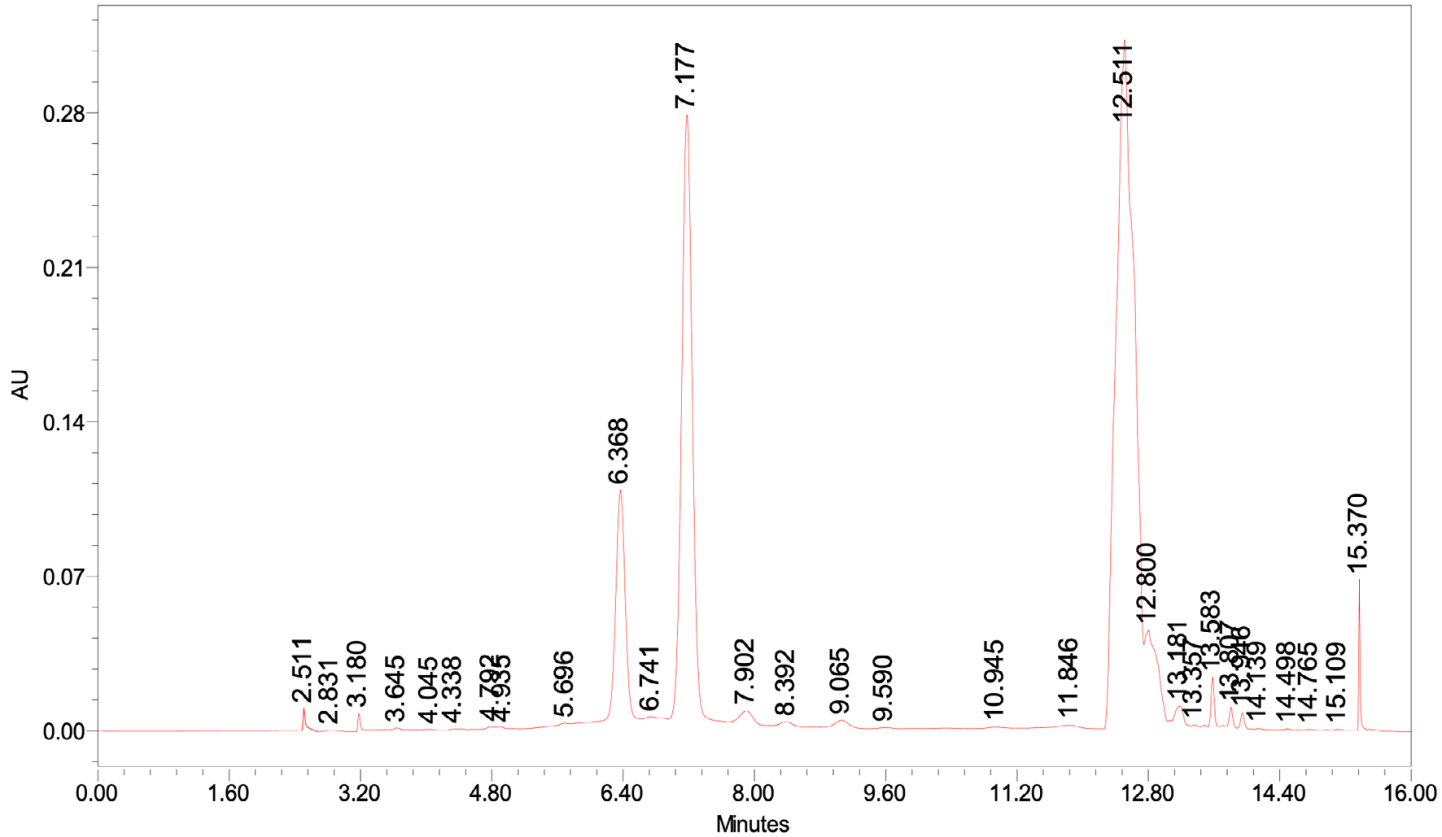
Representative HPLC chromatogram of the sappanwood (*Caesalpinia sappan* L.) extract. The chromatogram shows the overall chemical profile of the extract, with major peaks detected at retention times of 7.177.

**FIGURE 2:**
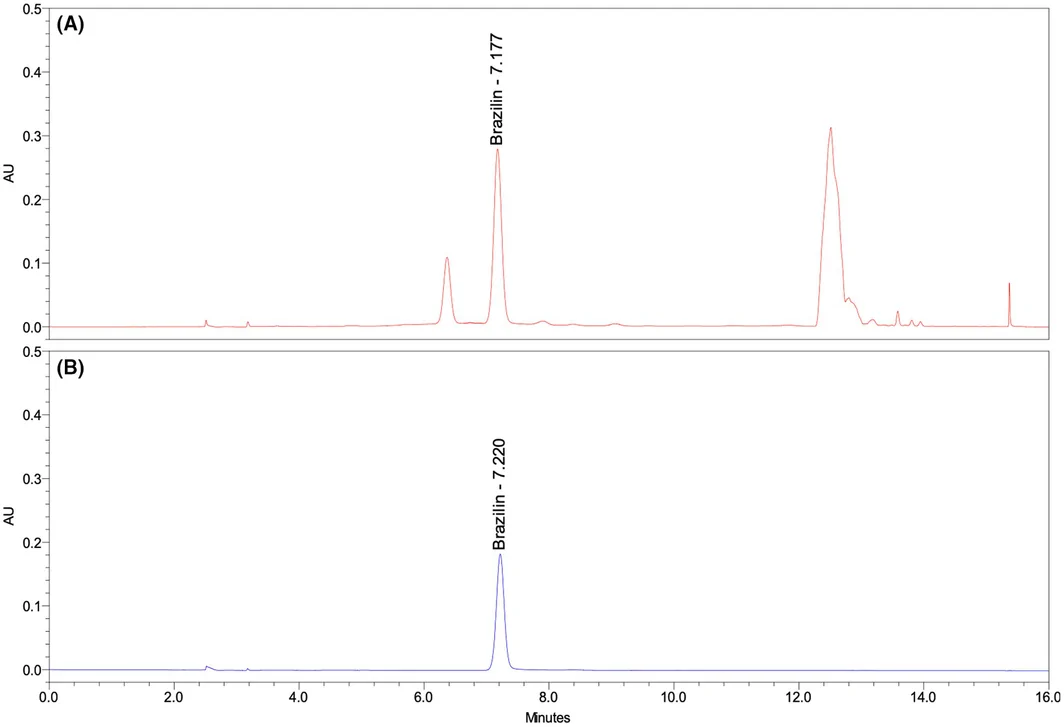
HPLC chromatograms. (A) Sappan wood (*Caesalpinia sappan* L.) extract. (B) Brazilin standard. The extract exhibited a prominent peak with a retention time similar to that of the brazilin standard.

The reagents used in this study included RNA extraction reagents (Quick‐RNA MiniPrep Plus; R1057‐Zymo Research, USA), cDNA synthesis reagents (SensiFAST cDNA Synthesis Kit, BIO‐98005; Bioline, UK), and gene‐specific primers (Macrogen, South Korea) for molecular analysis. Reagents for biochemical assays, including bilirubin (direct, indirect, and total), SGPT (ALT), and SGOT (AST), were obtained from Glory Diagnostic. Histological staining reagents, such as hematoxylin–eosin and absolute ethanol, were purchased from Merck (Germany). Reagents for the TBARS assay included phosphate‐buffered saline (PBS), potassium chloride (KCl), hydrochloric acid (HCl), trichloroacetic acid (TCA), ammonium thiocyanate, and butylated hydroxytoluene (BHT). Digestion reagents for total iron analysis with atomic absorption spectroscopy (AAS) included nitric acid (HNO_3_) and hydrogen peroxide (H_2_O_2_). Other chemicals included a standardized sappanwood extract (*Caesalpinia sappan* L.), prepared and standardized by PT Berkah Alam Indonesia. HPLC‐PDA analysis identified brazilin as the marker compound with a retention time of 7.177 min (Figures 1 and 2). The extract contained 25.127% ± 0.3868% brazilin (Table 1), and phenylhydrazine (Merck, Germany).

**TABLE 1:** HPLC‐PDA standardization results of the sappan wood (*Caesalpinia sappan* L.) extract used in this study, including brazilin content, linearity, limit of detection (LoD), and limit of quantification (LoQ).

Batch	Marker compound	Detector	Brazilin content (mg/mg extract) ± SD	Brazilin content (%) ± SD	LoD (μg/mL)	LoQ (μg/mL)	Regression equation (*r* ^2^)
2	Brazilin	PDA	0.2512 ± 0.0039	25.127 ± 0.3868	0.2986	0.9048	*Y* = 13 261*x* + 189.17 (0.9998)

### 2.2 Animals

The study used 24 male Wistar rats (180–250 g), obtained from PT Biofarma. Following a 7‐day acclimatization period, eligible animals were randomly allocated to six experimental groups (*n* = 4 per group) using a computer‐generated randomization sequence created in Microsoft Excel to minimize selection bias. The groups consisted of a control group, a phenylhydrazine (PHZ)‐induced hemolytic anemia group, three groups receiving different doses of sappanwood extract, and a deferiprone‐treated group. Only healthy animals were included in the study; rats exhibiting signs of illness, severe stress, or body weight loss exceeding 10% during acclimatization were excluded. Animals were housed at the Sitohistotechnology Laboratory, Universitas Jenderal Achmad Yani, in plastic cages with wire lids and husk bedding, which was replaced twice weekly. Environmental conditions were maintained at 25 ± 2°C with 30%–60% relative humidity under a 12‐h light–dark cycle. All experimental procedures involving animals were reviewed and approved by the Institutional Animal Care and Use Committee of Universitas Padjadjaran, under protocol number 414/UN6.KEP/EC/2025. All animal handling complied with the guidelines for the care and use of laboratory animals. Animals were monitored daily for signs of pain, distress, abnormal behavior, reduced mobility, or substantial weight loss. Predefined humane endpoints included severe illness, persistent distress, inability to access food or water, or excessive weight loss. Animals meeting these criteria would have been humanely euthanized to prevent unnecessary suffering.

### 2.3 Treatment and preparation

Test materials included phenylhydrazine (PHZ), sapanwood extract (SWE), and deferiprone, all dissolved in 0.9% NaCl. SWE and deferiprone were given orally through gavage, while PHZ was injected intraperitoneally for 2 days and given again after 7 days. The PHZ dose was set to the effective anemia‐inducing dose of 40 mg/kg BW,^22^ and doses were adjusted based on daily body weight. SWE was administered for 14 consecutive days at 50, 100, and 200 mg/kg BW. Deferiprone was administered orally at 25 mg/kg BW per dose, three times daily (total daily dose: 75 mg/kg BW) and served as the positive control treatment. The selected dose was based on previously validated experimental protocols in rats, while the dosing frequency was maintained in accordance with the Guidelines for the Clinical Management of Thalassemia (2nd Revised Edition) to mimic the clinical therapeutic schedule. The selected SWE doses (50, 100, and 200 mg/kg BW) were based on previous studies investigating the biological effects of sappan wood extract in rat models. These doses were chosen to represent a range of concentrations previously reported to exhibit biological activity and were used to evaluate potential dose‐related responses in the present study.^23^

At the end of the experimental period, rats were anesthetized with an intraperitoneal injection of ketamine (50 mg/kg BW) and xylazine (8 mg/kg BW). The dosing regimen was selected based on the recommended anesthetic ranges for laboratory rats described in Laboratory Animal Anesthesia (3rd edition). Adequate depth of anesthesia was confirmed by the absence of pedal withdrawal and corneal reflexes. Euthanasia was subsequently performed by exsanguination via cardiac puncture, followed by laparotomy for organ collection, in accordance with the AVMA Guidelines for the Euthanasia of Animals (2020) and institutional guidelines for the care and use of laboratory animals.

### 2.4 Organ collection, morphometric measurement, and histopathological analysis

After completion of the experimental protocol, animals were anesthetized using a combination of ketamine and xylazine administered intraperitoneally, and liver tissues were collected and weighed using an analytical balance to evaluate the effect of SWE on hepatomegaly. Livers were rinsed with physiological saline before the absolute organ weight was determined. Relative liver weight was then calculated using the formula: Organ Index = (Absolute organ weight/Body weight) × 100%. After weighing, the organs were sectioned for biochemical analysis and gene expression. For histology, after the organs were processed using the paraffin block method, the staining procedure began with deparaffinization in absolute xylene (two cycles, 10 min each), followed by graded rehydration in ethanol (100%, 90%, 80%, 70%) and distilled water (1 min each). Sections were stained with Harris hematoxylin for 20 min, rinsed in running water, differentiated in 0.25% lithium carbonate, and then stained with 1% eosin for 10 min. Dehydration was performed using graded ethanol (70%–100%), followed by absolute ethanol (1:1) and xylene (two cycles, 5 min each). The sections were then mounted on slides for microscopic evaluation. The Prussian blue staining process began with a deparaffinization step, with the slide being immersed three times in absolute xylene solution for 4 min each. The slide was rehydrated through a series of graded ethanol solutions, starting with absolute ethanol, then 90%, 80%, and finally 70%, for 4 min each, followed by a wash under running water. The next step was to incubate the slide in a 2% potassium ferrocyanide (II) solution at a 1:1 ratio with 2% hydrochloric acid (HCl) solution for 20 min. This incubation allows visualization of hemosiderin deposits in the tissue, which form a characteristic blue ferric‐ferrocyanide complex. To minimize assessment bias, histopathological evaluations, including hepatic hemosiderin aggregation and extramedullary hematopoiesis scoring, were conducted by an investigator blinded to treatment allocation.

### 2.5 Biochemical and tissue iron analysis

Serum bilirubin analysis included total and direct bilirubin, while indirect bilirubin was calculated as the difference between total and direct values (Indirect bilirubin = Total bilirubin − Direct bilirubin). Blood was collected using standard phlebotomy equipment and centrifuged to obtain plasma. Measurements were performed using the Erba XL‐200 based on spectrophotometric principles following the Glory Diagnostics kit protocol (GD‐BD100 and GD‐BD200, Spain). Briefly, 1.0 mL working reagent was added to labeled tubes (blank, standard, sample), followed by 100 μL distilled water, calibrator, or plasma, respectively. After mixing and incubating for 2 min at room temperature, absorbance was measured at 540 nm using a reagent blank as a reference. Color stability was maintained for up to 60 min.

The SGPT and SGOT procedures were performed according to the instructions for the Glory Diagnostics kit (GD‐GOT100 and GD‐GPT100 Spain). Working reagent (1 mL) was mixed with the sample (50 μL) at 37°C until homogeneous, then pipetted into a clean cuvette. The cuvette containing the reagent was placed in the spectrophotometer as a blank, and the initial absorbance was recorded at 340 nm. After blank measurement, the cuvette was removed, and the sample or control was added, mixed gently, and immediately reinserted into the spectrophotometer. Absorbance was recorded immediately (minute 0) and every minute for three consecutive minutes. As this is a kinetic method, SGPT and SGOT activities were calculated from the change in absorbance per minute (Δ A/min) at 340 nm, which reflects the decrease in Nicotinamide Adenine Dinucleotide (NADH). The reaction was considered valid if the absorbance change per minute was consistent and stable.

The malondialdehyde measurement procedure was performed using approximately 1.25 g of fresh liver, minced on ice in 2.5 mL of PBS containing 11.5 g/L KCl. The homogenate was centrifuged at 4000 rpm for 10 min, after which 0.5 mL of sample or standard was then added to 2 mL of a cold 0.25 N HCl mixture containing 15% trichloroacetic acid (TCA), 0.38% thiocyanic acid (TBA), and 0.5% butyl hydroxytoluene (BHT). The mixture was heated at 80°C for 1 h. After cooling, the sample and standard mixture was centrifuged at 3 500 rpm for 10 min. The absorbance of the supernatant was then measured at 532 nm, using 1,1,3,3‐tetraethoxypropane (TEP) as the standard.

For the total iron liver measurement procedure, liver tissue samples (0.2 g) were placed in a 100 mL beaker, then 5 mL HNO_3_ was added. The mixture was heated until tissue shrinkage occurred, then 2 mL of 30% H_2_O_2_ was added and the mixture was heated further until gas bubbles appeared. After digestion, the solution was diluted to 25 mL in a volumetric flask and homogenized. Iron concentration was measured using atomic absorption spectroscopy (AAS) at 248.3 nm. The Fe concentration was calculated using the formula:

$$\mathrm{Fe}\;\text{concentration}\;\left(\mathrm{mg}/g\;\text{tissue}\right)=\left(C\times V\right)/W$$

where *C* is the Fe concentration measured by AAS (mg/L), *V* is the final volume after digestion (L), and *W* is the tissue weight (g).

### 2.6 RNA synthesis and cDNA preparation

Total RNA was retrieved using the protocol from the Quick‐DNA/RNA Miniprep Plus Kit (Zymo Research, California, USA) Samples lysed in RNA lysis buffer were transferred to Spin‐Away™ Filters in collection tubes and centrifuged to remove genomic DNA. The flow‐through was mixed 1:1 with 95%–96% ethanol, homogenized, and applied to Zymo‐Spin™ IIICG columns, then centrifuged. Initial washing was performed with 400 μL RNA wash buffer. Then, 40 μL DNase I (1 U/μL) mixed with 600 μL DNA Digestion Buffer was added to the column and incubated at room temperature for 15 min. After adding 400 μL RNA Prep Buffer, sequential washes were performed with 700 μL and 400 μL RNA wash buffer. A final wash with RNA wash buffer was done, followed by 1‐min centrifugation. The column was transferred to a nuclease‐free tube, and RNA was eluted with 50 μL RNase‐free water. The resulting cDNA was quantified using a spectrophotometer Tecan Infinite® 200 PRO (Tecan Group Ltd., Männedorf, Switzerland).

cDNA synthesis was performed using the SensiFAST cDNA Synthesis Kit (Bioline, London, UK) following the manufacturer's instructions. All reagents were kept on ice, vortexed, and briefly centrifuged before use. The reaction mix was prepared by combining 1 μg of total RNA or mRNA, 4 μL of 5× TransAmp Buffer, and 1 μL of Reverse Transcriptase, then adding DNase/RNase‐free water to a final volume of 20 μL. The mixture was gently homogenized with a pipette. cDNA synthesis was carried out on a thermal cycler with the following program: 25°C for 10 min for primer annealing, followed by 42°C for 15 min for reverse transcription. The resulting cDNA was quantified using a spectrophotometer (Tecan).

### 2.7 Quantitative PCR (qPCR)

PCR reactions were performed using the SensiFAST SYBR® No‐ROX Kit (Bioline, London, UK). The 20 μL reaction mix consisted of 10 μL 2× SensiFAST SYBR® No‐ROX Mix, 0.8 μL forward primer (10 μmol/L), 0.8 μL reverse primer (10 μmol/L), template cDNA up to 8.4 μL, and nuclease‐free water to a final volume of 20 μL. The Primer sequence used is: Bone Morphogenetic Protein 6 *(Bmp‐6)* = *Bmp*‐6 Forward 5′‐GCTCCAGTGCTTCAGACTAC‐3′ *Bmp‐6* Reverse 5′‐GATGATCCAGTCCTGCCATC‐3′ Hemojuvelin (*Hjv)* = *Hjv* Forward 5′‐TGCAGCCTTTGAAGATGGTT‐3′ *Hjv* Reverse 5′ ‐TGTTCCAATGTAGGCAGCTC‐3′ Hepcidin Antimicrobial Peptide *(Hamp)* = *Hamp* Forward 5′‐ACAGACGAGACAGACTACGG‐3′ *Hamp* Reverse 5′‐GAGGCATATGGGGAAGTTGG‐3′ Erythropoietin *(Epo)* = *Epo* Forward 5′‐TCTGACTGACCGGTTTACTC‐3′ *Epo* Reverse 5′‐GCCCAGAGGAATCAGTAGCA‐3′ Ferritin Light Chain = *(Ftl) Ftl Forward Ftl Revers*‐F: GCTGGCTTCTTGATGTCC R: CCTCCTACACCTACCTCTC.^24^, ^25^, ^26^, ^27^ PCR optimization was performed prior to gene expression analysis to ensure reliable amplification. Amplification efficiency for each primer pair was evaluated using a standard curve generated from serial dilutions of cDNA templates. The calculated PCR efficiency ranged from 93% to 98%.

### 2.8 Statistical analysis

Statistical analyses were performed using Statistical Package for the Social Sciences (SPSS) version 21.0 (IBM Corp., Armonk, NY, USA). Quantitative data obtained from biochemical measurements, organ weight assessments, and ImageJ‐based image analyses were expressed as mean ± standard deviation (SD). Prior to inferential analysis, the assumptions for parametric testing were evaluated. Data normality was assessed using the Shapiro–Wilk test, while homogeneity of variances among groups was evaluated using Levene's test. As all quantitative datasets met the assumptions of normality and homogeneity of variance (*p* > 0.05), intergroup differences were analyzed using one‐way analysis of variance (ANOVA). When a significant overall effect was detected, Tukey's Honestly Significant Difference (HSD) test was applied for multiple pairwise comparisons. Data that did not satisfy the assumptions of normality and/or homogeneity of variance would have been analyzed using the non‐parametric Kruskal–Wallis test. Histopathological parameters, including hepatic hemosiderin aggregation scores in Prussian blue‐stained sections and extramedullary hematopoiesis scores in hematoxylin and eosin (H&E)‐stained liver sections, were treated as ordinal data and therefore analyzed using non‐parametric methods. Differences among groups were evaluated using the Kruskal–Wallis test, followed by pairwise Mann–Whitney *U* tests for post hoc comparisons when appropriate. A *p*‐value of less than 0.05 was considered statistically significant for all analyses.

## 3 RESULTS

### 3.1 Alterations in relative liver weight, hemosiderin aggregation, and extramedullary hematopoiesis following PHZ induction and Sappanwood extract administration

**FIGURE 3:**
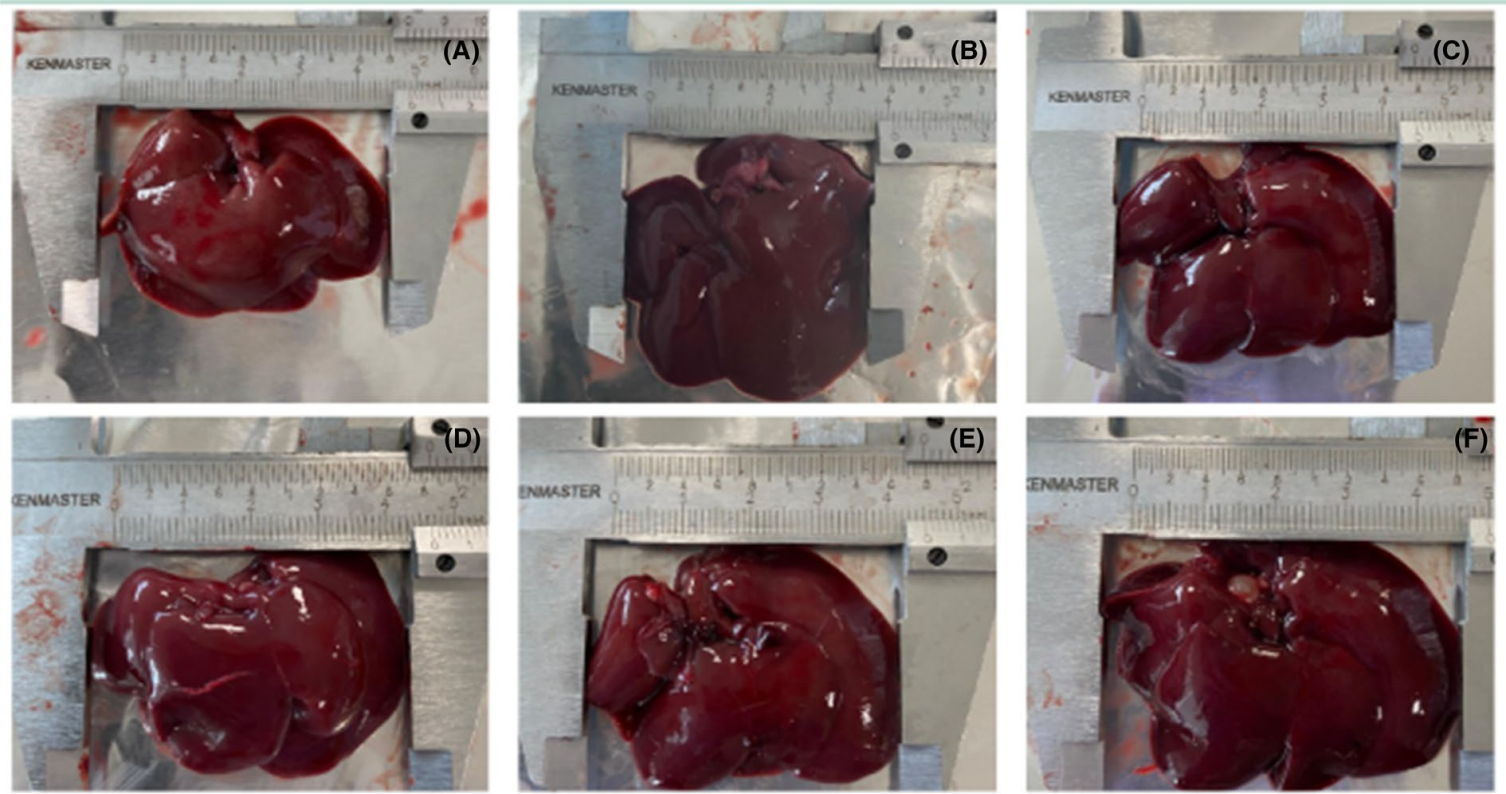
Morphological appearance of rat liver in different treatment groups. (A) Normal rat; (B) PHZ induced hemolytic anemia; (C) PHZ + DFP; (D) PHZ + SWE 50 mg/kg; (E) PHZ + SWE 100 mg/kg; (F) PHZ + SWE 200 mg/kg.

**FIGURE 4:**
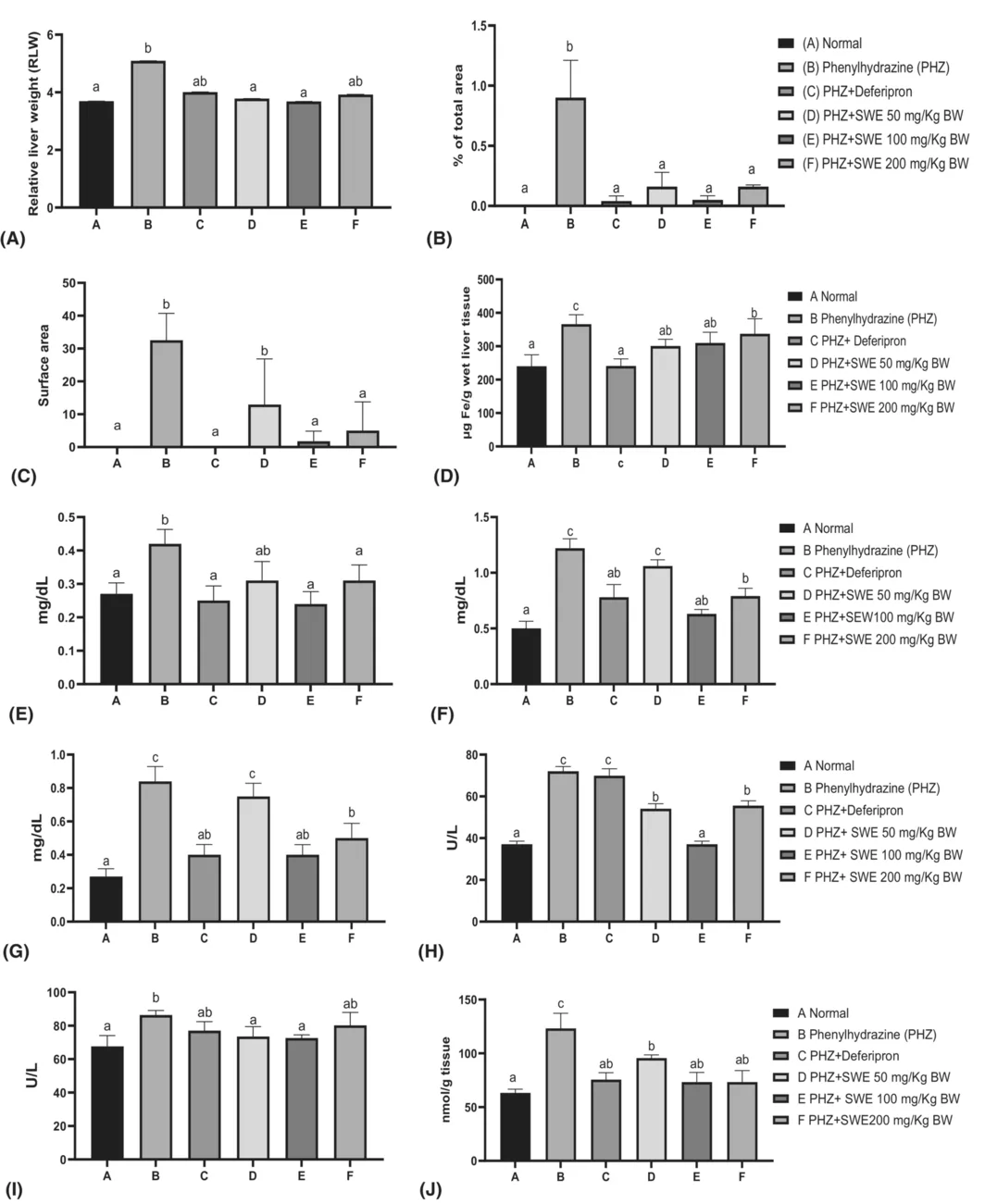
Effects of PHZ, DFP, and SWE on hepatic and hematologic parameters in rats. (A) Liver weight. (B) Hepatic hemosiderin score. (C) Extramedullary hematopoiesis. (D) Hepatic iron concentration measured by atomic absorption spectrophotometry. (E–G) Bilirubin fractions. (H) Alanine aminotransferase (SGPT). (I) Aspartate aminotransferase (SGOT). (J) Hepatic malondialdehyde (MDA). Biochemical parameters were analyzed using one‐way ANOVA followed by Tukey's post hoc test (α = 0.05). Histopathological scoring parameters were analyzed using the non‐parametric Kruskal‐Wallis test followed by appropriate post hoc comparisons. Different letters above bars indicate significant differences (𝑝 < 0.05), while identical letters indicate non‐significant differences.

**FIGURE 5:**
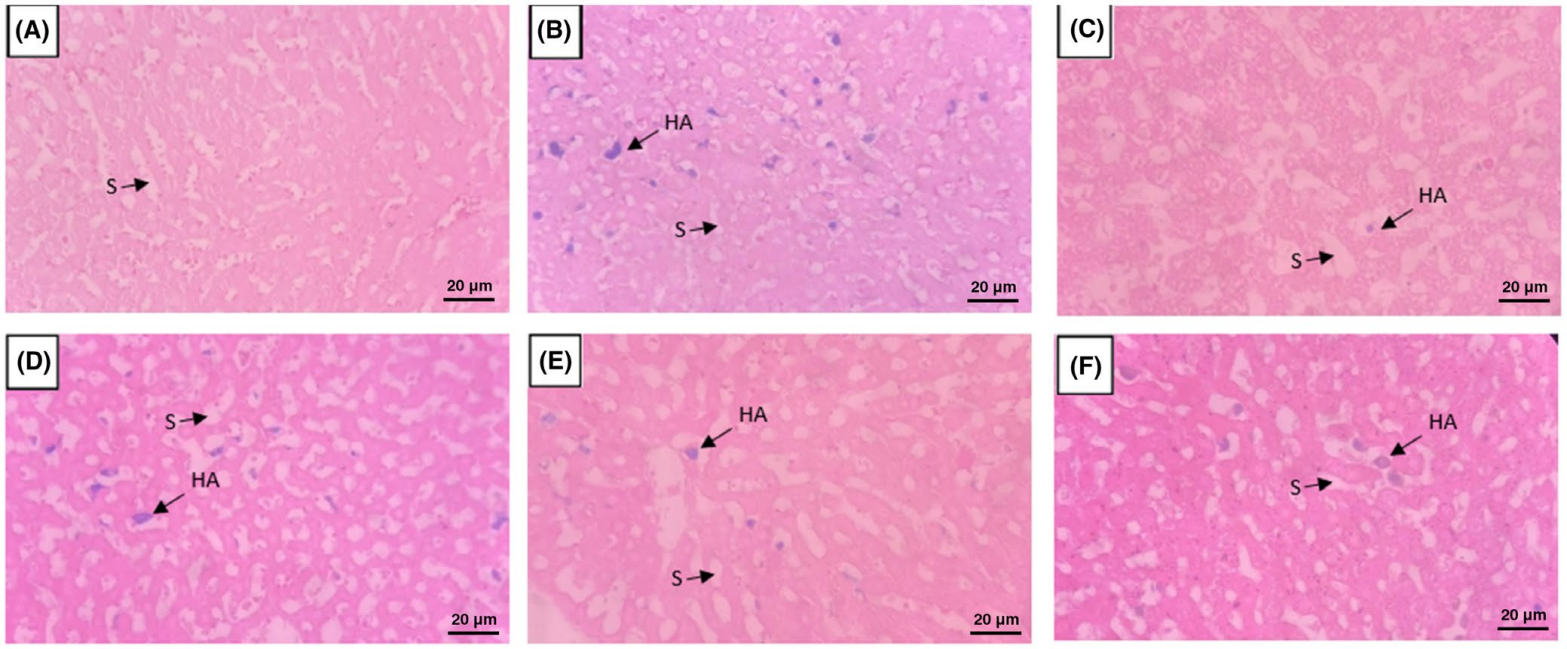
Histopathological features of rat liver stained with Prussian blue (400×). (A) Normal rat, (B) PHZ‐treated rat, (C) DFP‐treated rat, (D) PHZ + SWE (50 mg/kg), (E) PHZ + SWE (100 mg/kg), (F) PHZ + SWE (200 mg/kg). HA, hemosiderin aggregates; S, sinusoid.

**FIGURE 6:**
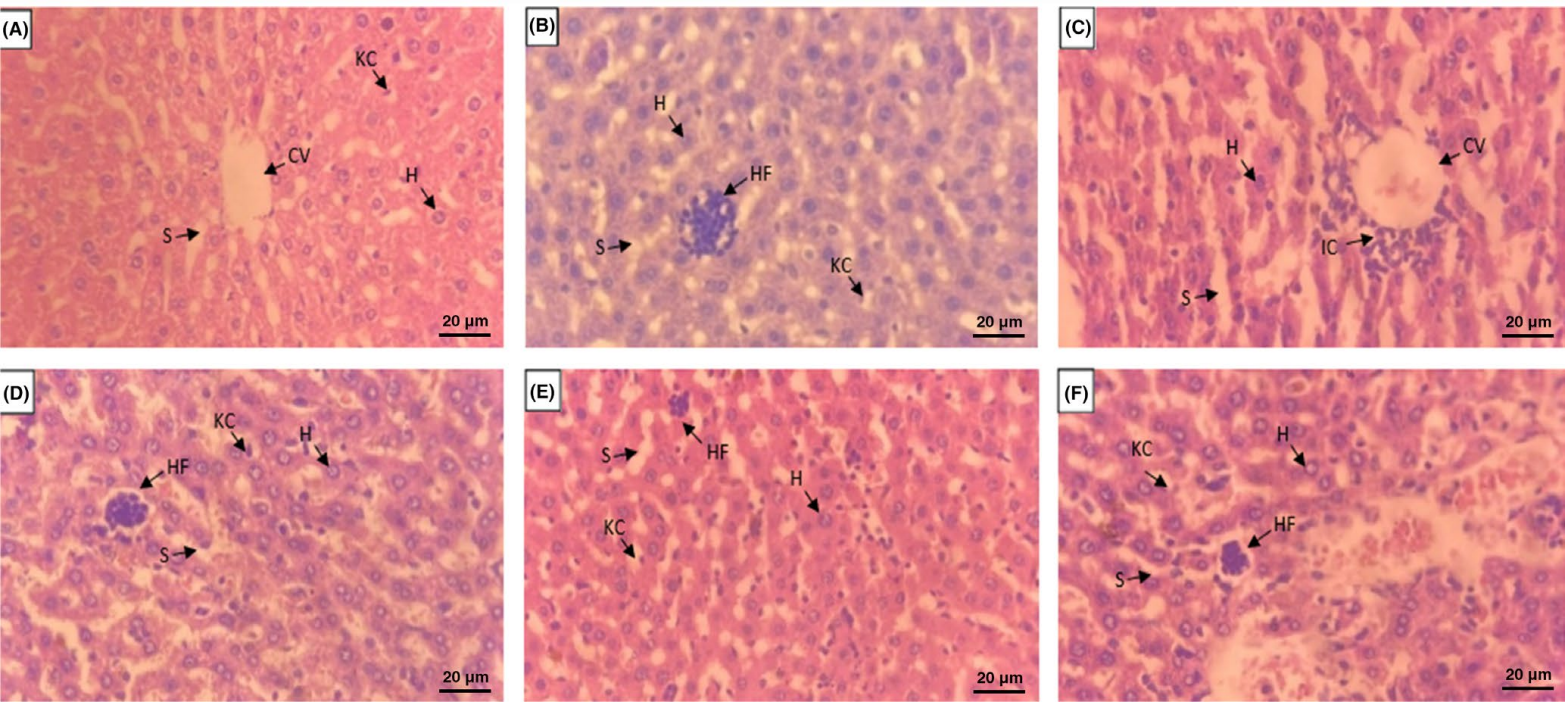
Histopathological features of rat liver stained with H&E (400×). (A) Normal rat, (B) PHZ‐treated rat, (C) DFP‐treated rat, (D) PHZ + SWE (50 mg/kg), (E) PHZ + SWE (100 mg/kg), (F) PHZ + SWE (200 mg/kg). CV, central vein; H, hepatocyte; HF, hematopoietic focus; IC, inflammatory cell; KC, Kupffer cell; S, sinusoid.

Rats treated with PHZ at 40 mg/kg BW showed a significant 38% increase in relative liver weight (*F* = (5,18) = 3.987, *p* = 0.013) compared with the standard control, indicating hepatomegaly following induction. Treatment with DFP and SWE at 200 mg/kg BW reduced liver weight by 21% and 23%, respectively, relative to the PHZ group but the relative weights did not differ significantly from either the standard control or the PHZ group (Figures 3c,f and 4a; Table 2). Meanwhile, SWE at doses of 50 and 100 mg/kg BW significantly reduced relative liver weight compared with the PHZ group, with decreases of 26% and 28%, respectively, and showed no significant difference from the standard control (Figures 3d‐e and 4a; Table 2). Administration of PHZ at 40 mg/kg BW resulted in a significant increase (*H* = (5) =18.481, *p* = 0.002) in Prussian blue staining, compared with the normal control group, indicating an elevation in the total hepatic hemosiderin aggregate area because of iron overload following hemolysis (Figure 5b; Table 2). Treatment with DFP at 75 mg/kg BW also showed a significant difference compared with both the normal and PHZ groups, with a 96% reduction in total hemosiderin area, demonstrating a marked effect of DFP in alleviating hepatic iron overload. Meanwhile, treatment with SWE at doses of 50, 100, and 200 mg/kg BW resulted in significant reductions in total hemosiderin aggregate area compared with the PHZ group—by 82%, 82%, and 94%, respectively—with the 100 mg/kg BW dose providing the most optimal reduction (Figures 4b and 5c–f; Table 2). Administration of PHZ at 40 mg/kg BW resulted in a significant increase (*H* = (5) =14.847, *p* = 0.011) in H&E staining, compared with the normal control group, indicating an expansion of the hepatic extramedullary hematopoiesis (EMH) area because of hemolysis induction (Figure 6b; Table 2). Treatment with DFP at 75 mg/kg BW also showed significant differences in staining compared with both the normal and PHZ groups, with a complete (100%) reduction in EMH area, suggesting that DFP markedly suppresses extramedullary hematopoietic activity (Figures 4c and 6c; Table 2). Meanwhile, SWE exhibited a dose‐dependent effect; the 50 mg/kg BW dose did not produce a significant difference in staining, showing only a 60% reduction, whereas the 100 and 200 mg/kg BW doses significantly reduced the hepatic EMH area compared with the PHZ group, achieving reductions of 95% and 84%, respectively (Figures 4c and 6d–f; Table 2).

**TABLE 2:** Biochemical, histopathological, and morphometric parameters of the liver.

Parameters	Normal	PHZ	DFP	SWE 50	SEW 100	SWE 200
Relative liver weight (%)	3.69 ± 0.001	5.09 ± 0.001	4.01 ± 0.004	3.78 ± 0.005	3.68 ± 0.003	3.92 ± 0006
SGPT (U/L)	37.07 ± 1.508	72.04 ± 2.219	69.88 ± 3.351	54.12 ± 2.367	37.08 ± 1.508	55.56 ± 2.335
SGOT (U/L)	67.66 ± 6.496	86.38 ± 2.714	77.07 ± 5.367	73.53 ± 5.928	72.64 ± 1.864	80.23 ± 7.691
Indirect bilirubin (mg/dL)	0.27 ± 0.047	0.84 ± 0.089	0.40 ± 0.062	0.75 ± 0.079	0.38 ± 0.061	0.53 ± 0.089
Direct bilirubin (mg/dL)	0.24 ± 0.003	0.79 ± 0.043	0.53 ± 0.044	0.75 ± 0.057	0.037 ± 0.037	0.31 ± 0.047
Total bilirubin (mg/dL)	0.50 ± 0.064	1.22 ± 0.086	0.78 ± 0.114	1.06 ± 0.057	0.63 ± 0.041	0.79 ± 0.071
EMH (μm^2^)	0.00 ± 0.00	32.54 ± 8.178	0 ± 0	8.29 ± 13.92	1.77 ± 3.066	5.04 ± 5.04
Hemosiderin aggregates (%)	0.00 ± 0.00	0.90 ± 0.331	0.04 ± 0.043	0.16 ± 0.119	0.05 ± 0.034	0.16 ± 0.015
Malondialdehyde (nmol/g tissue)	63.13 ± 3.455	123.04 ± 14.06	75.43 ± 6.527	95.42 ± 2.990	73.20 ± 8.934	73.20 ± 10.634
Total liver iron content (μg Fe/g wet liver tissue)	240 ± 34.25	366 ± 28.23	241 ± 21.12	300 ± 20.08	309 ± 32.29	337 ± 44.94

*Note*: Values are expressed as mean ± SD (*n* = 4 per group).

Abbreviations: DFP, PHZ+ deferiprone; EMH, extramedullary hematopoiesis; MDA, malondialdehyde; NORMAL, normal control; PHZ, phenylhydrazine; SGOT, serum glutamate oxaloacetate transaminase (AST); SGPT, serum glutamate pyruvate transaminase (ALT); SWE 50, SWE 100, SWE 200, PHZ + sappan wood extract at doses of 50, 100, and 200 mg/kg body weight, respectively.

### 3.2 Biochemical alterations following PHZ‐induced hemolysis and Sappanwood extract administration: Changes in bilirubin levels, liver enzymes, oxidative stress, and hepatic iron

Administration of phenylhydrazine (PHZ) at 40 mg/kg BW resulted in a significant increase (*F* = (3.18) =20.064, *p* = <0.001) in indirect bilirubin, which rose by 207% compared with the standard control. Treatment with DFP and SWE at 100 and 200 mg/kg BW significantly reduced indirect bilirubin levels, with decreases of 41%, 55%, and 37%, respectively, compared with the PHZ group (Figure 4; Table 2). In contrast, SWE at 50 mg/kg BW did not produce a significant effect, showing only a 10% reduction. PHZ administration also resulted in a substantial increase in direct bilirubin (*F* = (5.18) =6.539, *p* = 0.001), with a 55% increase compared with the standard control and values significantly higher than those observed in the DFP and the 100 and 200 mg/kg BW SWE groups (Figure 4; Table 2). In contrast, SWE at 50 mg/kg BW did not differ significantly from either the standard control or the PHZ groups, resulting in only a 5% reduction. DFP and SWE at 100 and 200 mg/kg BW reduced direct bilirubin levels by 33%, 51%, and 40%, respectively. Similarly, PHZ at 40 mg/kg BW caused a significant increase in total bilirubin (*F* = (5.18) *p* = <0.001), which reached 145% above the standard control. The 50 mg/kg BW SWE group did not differ significantly from the PHZ group, showing only a 13% reduction. In contrast, SWE at 100 and 200 mg/kg BW decreased total bilirubin by 49% and 36%, respectively, whereas DFP reduced it by 36% compared with the PHZ group (Figure 4; Table 2).

Administration of PHZ at 40 mg/kg BW significantly increased SGPT levels (*F* = (5.18) =130.674, *p* = <0.001) by 94% compared with the standard control. The group treated with DFP at 75 mg/kg BW did not differ significantly from the PHZ group, with only a 3% reduction in SGPT levels, which remained substantially higher than the normal group. Sappanwood extract (SWE) at 50, 100, and 200 mg/kg BW produced significant decreases in SGPT levels compared with the PHZ group, with reductions of 25%, 49%, and 23%, respectively; however, the 50 and 200 mg/kg BW doses also showed significant differences from the standard control (Figure 4h; Table 2). Similarly, administration of PHZ at 40 mg/kg BW resulted in a substantial 28% increase in SGOT levels (*F* = (5.18) =4.941, *p* = 0.005), compared with the standard control. The group treated with DFP at 75 mg/kg BW did not differ significantly from either the PHZ or the normal groups, although DFP reduced SGOT by 11% relative to PHZ. Treatment with SWE at 50 and 100 mg/kg BW resulted in significant reductions in SGOT levels of 15% and 16%, respectively, compared with the PHZ group. In contrast, the group treated with 200 mg/kg BW dose did not differ significantly from either of the normal or PHZ groups (Figure 4i; Table 2).

Administration of PHZ at 40 mg/kg BW resulted in a significant 95% increase in MDA levels (*F* = (5.18) =19.004, *p* = <0.001) compared with the normal control group. The mean MDA concentration in the PHZ group was 123 nmol/g tissue, whereas in the normal group it was only 63.13 nmol/g tissue. This increase indicates that PHZ induction successfully triggered oxidative stress due to hemolysis and the accumulation of free iron in hepatic tissue. In contrast, treatment with deferiprone (75 mg/kg BW) and sappanwood extract (SWE) at doses of 50, 100, and 200 mg/kg BW significantly reduced MDA levels compared with the PHZ group, with reductions of 39% (DFP), 22% (SWE 50), 41% (SWE 100), and 22% (SWE 200) (Figure 4j; Table 2). Administration of PHZ at 40 mg/kg BW significantly increased total hepatic iron levels by 41% compared with the standard control. The mean total iron concentration in the PHZ group was 366 mg/kg tissue, whereas in the normal group it was 240 mg/kg tissue. This marked increase reflects iron accumulation resulting from PHZ‐induced hemolysis, in which erythrocyte membrane disruption leads to cell rupture and the subsequent release of hemoglobin‐derived iron into the liver. Treatment with deferiprone (DFP) at 75 mg/kg BW significantly reduced hepatic total iron levels compared with the PHZ group, which decreased from 366 mg/kg to 241 mg/kg tissue, corresponding to a 35% reduction. Similarly, SWE at 50, 100, and 200 mg/kg BW significantly reduced hepatic total iron levels, with levels decreasing from 366 mg/kg to 300, 309, and 337 mg/kg, respectively. These reductions correspond to decreases of 15% (50 mg/kg), 12% (100 mg/kg), and 11% (200 mg/kg) relative to the PHZ group (Figure 4d; Table 2).

### 3.3 Modulation of iron regulatory gene expression following PHZ‐induced hemolysis and Sappanwood extract treatment

**FIGURE 7:**
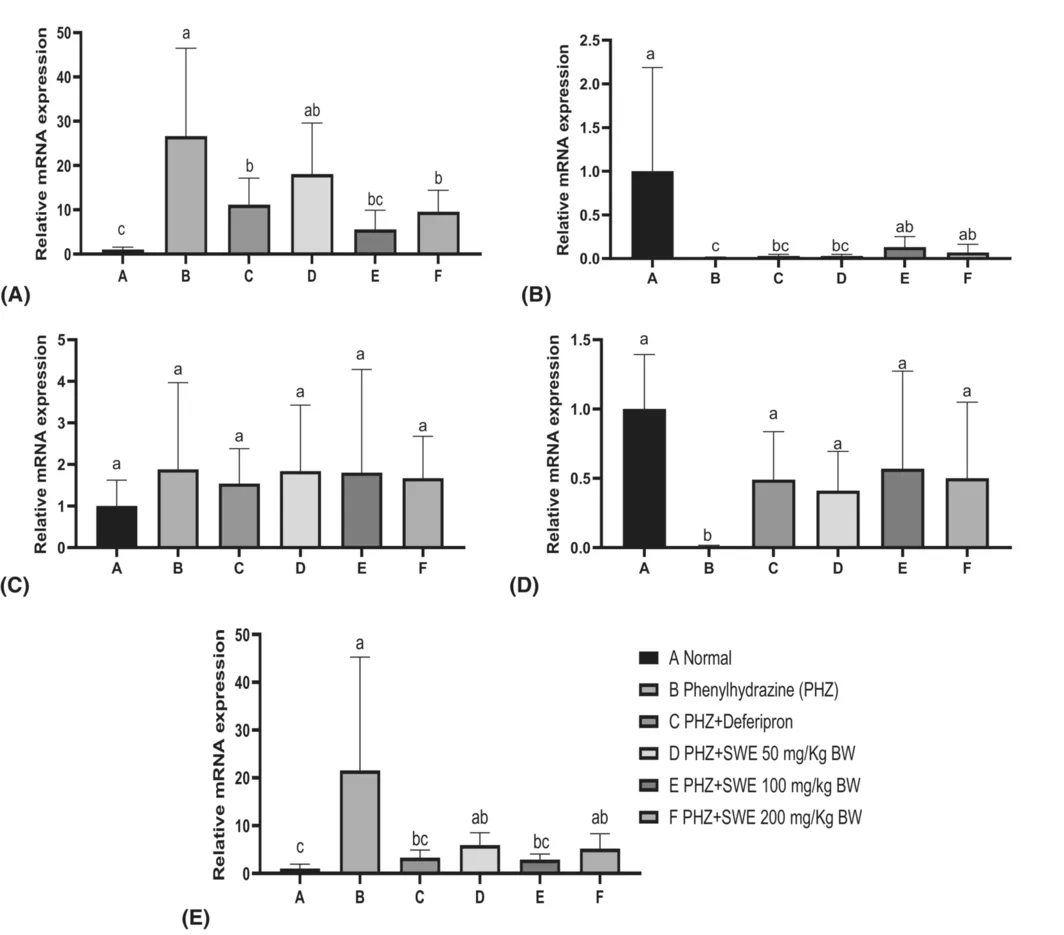
Effects of PHZ, DFP, and SWE on hepatic gene expression in rats. Panels show relative mRNA expression. (A) Erythropoietin (*Epo*). (B) Bone morphogenetic protein 6 *(Bmp‐6*). (C) Hemojuvelin *(Hjv)*. (D) Hepcidin *(Hamp*). (E) Ferritin light chain *(Ftl)*. Statistical analysis was performed using one‐way ANOVA followed by Tukey's post hoc test (α = 0.05). Different letters above bars indicate significant differences (𝑝 < 0.05), while identical letters indicate non‐significant differences.

qPCR analysis demonstrated that PHZ induction led to a marked, nearly 100‐fold, downregulation of *Bmp‐6* expression (*F* = (5,18) =8.152, *p* < 0.001). Treatment with DFP (75 mg/kg BW) produced only a modest recovery, with expression remaining 33‐fold downregulated, indicating that DFP did not fully reverse the PHZ‐induced suppression. In contrast, SWE at 100 and 200 mg/kg BW markedly restored *Bmp‐6* expression, reducing the deficit to only 7‐fold and 14‐fold, respectively, whereas the 50 mg/kg BW dose remained ineffective. PHZ induction also led to a severe downregulation of *Hamp* (*F* = (5,18) =9.639, *p* = <0.001), lowering expression to approximately 1% of normal levels. DFP significantly increased Hamp expression, limiting the PHZ‐induced reduction to 2‐fold compared with normal. SWE at 50, 100, and 200 mg/kg BW likewise significantly elevated *Hamp* expressions, with remaining PHZ‐induced reductions of 2.4‐, 1.75‐, and 2.4‐fold, respectively. Conversely, PHZ markedly upregulated *Ftl* (*F* = (5,18) =4.434, *p* = 0.08), resulting in a 21.56‐fold increase relative to normal levels. DFP effectively normalized *Ftl* expression, reducing it to 3.33‐fold above baseline. Among the SWE groups, only the 100 mg/kg BW dose significantly decreased *Ftl* expression compared with PHZ, yielding a 2.89‐fold increase relative to the normal level. In contrast, the 50 and 200 mg/kg BW doses resulted in 5.93‐ and 5.17‐fold increases, respectively. PHZ also triggered a significant increase in *Epo* expression (*F* = (5,18) =12.571, *p* ≤ 0.001), which reached 26.63‐fold above normal levels. DFP treatment reduced this response to 11.15‐fold, representing a considerable improvement. SWE at 50, 100, and 200 mg/kg BW also mitigated the PHZ‐induced increase, resulting in 18.6‐, 5.57‐, and 9.57‐fold increases in expression relative to control. Notably, only the 100 and 200 mg/kg BW doses produced statistically significant reductions, while 50 mg/kg BW showed no significant effect. In the treatment groups receiving 40 mg/kg BWPHZ, 75 mg/kg BW DFP, and 50, 100, and 200 mg/kg BW SWE, the RT‐PCR results and statistical analysis showed no significant differences between treatment groups (*F* = (5,18) = 0.590, *p* = 0.707). However, there was a slight increase in *Hjv* gene expression. This indicates that in hemolytic anemia, when BMP expression is suppressed, *Hjv* can still be expressed at near‐normal levels (Figure 7(a‐e)).

## 4 DISCUSSION

In this study, administration of phenylhydrazine (PHZ) at 40 mg/kg BW significantly increased relative liver weight, indicating hepatomegaly (Figures 3b, 4a, and 6b; Table 2). This increase was caused by PHZ‐induced erythrocyte hemolysis, which subsequently triggers hemolytic anemia and hypoxia. The mechanism involves the interaction between PHZ and hemoglobin, which induces oxidative processes. The formation of these radicals initiates a chain reaction that leads to erythrocyte membrane damage and sustained hemolysis. Persistent hemolytic anemia, accompanied by hypoxia, stimulates erythropoiesis and increases erythrocyte production. Under these conditions, the body activates the stress erythropoiesis mechanism, which then becomes the dominant pathway to meet the increased erythrocyte demand.^28^, ^29^ This effect makes PHZ a suitable model for hemolytic anemia and other hemolytic disorders in patients with thalassemia.

In prominent hemolysis where the bone marrow's capacity is insufficient to produce blood cells, the liver can resume its function as a site of blood cell formation, a condition known as extramedullary hematopoiesis. When extramedullary hematopoiesis (EMH) is active, blood‐forming cells begin to proliferate outside the bone marrow and occupy the sinusoidal spaces as well as the hepatic parenchyma. The accumulation of these hematopoietic cells increases the density and volume of liver tissue. This process explains why the relative liver weight in the PHZ group is higher than in the normal group (Figures 3b, 4b, and 6b; Table 2).^30^ Histological observations using Prussian blue staining revealed hemosiderin aggregates in the PHZ‐treated group, attributable to increased intracellular iron stores resulting from erythrocyte hemolysis. Excess iron saturates ferritin and is subsequently degraded in lysosomes, leaving Fe_2_O ions that precipitate and form insoluble iron oxide complexes known as hemosiderin (Figure 6b).^31^

Examination of bilirubin revealed elevated indirect bilirubin levels, confirming hemolysis. Following hemolysis, free hemoglobin binds to haptoglobin, forming a complex that is taken up by macrophages via the CD163 receptor and cleared from the plasma through the spleen or liver. The heme component of hemoglobin is then degraded by heme oxygenase‐1 (HO‐1) into free iron, carbon monoxide, and biliverdin, which is subsequently converted into indirect bilirubin.^32^, ^33^ The increase in direct bilirubin levels in the PHZ‐treated group is caused by a high hemolytic burden accompanied by extramedullary hematopoiesis (EMH) in the liver. EMH foci compress the hepatic parenchyma (Figure 6b), thereby reducing hepatocyte function and impairing bilirubin conjugation. Additionally, local inflammation from chronic EMH may obstruct bile flow, resulting in elevated direct bilirubin (Figure 4; Table 2). One of the rare complications of EMH is obstructive jaundice, which occurs when EMH masses block bile flow. This obstruction causes conjugated bilirubin to accumulate within hepatocytes and leak back into systemic circulation, resulting in direct hyperbilirubinemia.^34^ Total bilirubin reflects a combination of direct and indirect bilirubin. Examination of SGPT and SGOT revealed elevated levels of both (Figure 4h,i; Table 2). This elevation indicates that PHZ induction at this dose effectively enhances SGPT enzyme activity in experimental animals. The rise in SGPT is associated with hemolytic anemia, in which massive hemolysis releases excessive iron into the circulation, leading to iron overload and oxidative stress that can trigger hepatocellular damage and allow SGPT to leak into the bloodstream.^35^ A significant increase in SGOT levels was also observed in rats. This elevation is associated with liver injury from iron overload and erythrocyte membrane damage during hemolysis. However, it is also possible that part of the enzyme originates from other organs, as SGOT is not a specific marker of liver damage.^36^, ^37^

Moreover, hepatic malondialdehyde MDA levels were elevated due to PHZ‐mediated erythrocyte hemolysis, resulting iron overload (Figure 4j; Table 2). Under conditions of hepatic iron excess, iron‐catalyzed lipid peroxidation plays a key role in hepatocellular injury. MDA is the end‐product of the peroxidation reaction between free radicals and lipids and can directly reflect the degree of lipid oxidation. Iron is a significant promoter of ROS production, capable of initiating or catalyzing Fenton reactions that generate free radicals to damage liver cells. Iron overload leads to excessive ROS formation and increases intracellular calcium uptake. At the same time, ROS generated by iron overload further exacerbates lipid peroxidation and oxidative damage. This lipid peroxidation process produces reactive aldehydes, such as MDA and 4‐hydroxynonenal (4‐*HNE*), which can bind to proteins, forming protein–aldehyde adducts. The formation of these adducts can alter the structure and function of cellular proteins, thereby exacerbating cellular damage and dysfunction.^38^, ^39^, ^40^ Hemolysis also significantly increased total iron levels in the liver (Figure 4d; Table 2). PHZ‐induced hemolysis releases iron from erythrocytes into the circulation. Simultaneously, the body interprets this condition as a hypoxic signal, stimulating increased intestinal iron absorption to support erythropoiesis. However, under sustained hemolysis, this compensatory mechanism may ultimately lead to excessive iron accumulation. Iron overload is characterized by excessive iron deposition in various organs, particularly the liver, which is the primary site of iron storage and detoxification. Approximately 20% of the body's iron reserves are stored in the liver, while about 5% are found in tissue macrophages.^41^

Although the overall trends observed by Prussian blue staining and AAS were generally comparable, the magnitude of the changes was not always proportional. This difference likely reflects the distinct biological compartments measured by each method. Prussian blue staining primarily detects ferric iron deposited as hemosiderin within tissue sections and therefore provides information on localized iron storage deposits. In contrast, AAS quantifies total hepatic iron content in homogenized tissue samples regardless of its cellular localization or biochemical form. Consequently, AAS includes iron stored in ferritin, hemosiderin, heme‐containing proteins, and other intracellular iron pools, whereas Prussian blue staining mainly reflects hemosiderin accumulation. Therefore, changes in hemosiderin deposition may not necessarily correspond to proportional changes in total liver iron content. In addition, heterogeneous iron distribution within the liver and differences in methodological sensitivity may further contribute to discrepancies between histological observations and AAS‐based iron quantification. It is also possible that a fraction of iron chelated by brazilin remains temporarily retained within hepatic tissue before subsequent metabolism and excretion. Under such circumstances, total hepatic iron measured by AAS may remain relatively elevated despite a reduction in visible hemosiderin deposition. However, this interpretation remains speculative because the tissue distribution, metabolism, and elimination of brazilin–iron complexes were not directly evaluated in the present study.

In this study, PHZ‐induced hemolytic anemia and hypoxia increased hepatic *Epo* gene expression (Figure 7a). The expression of *Hif‐2*, which regulates hepatic *Epo*, is primarily determined by oxygen status and inflammatory cytokines (factors supporting EMH), rather than by iron status alone. These findings indicate that iron overload, as reflected by increased *ftl* expression, does not suppress but rather enhances hepatic *Epo* expression.^25^, ^42^ Moreover, *Bmp6* gene expression was decreased (Figure 7b,c). In this study, an increase in erythropoietin *(Epo)* levels was accompanied by a decrease in *Bmp6* expression, suggesting an inverse relationship between erythropoietic drive and *Bmp6*‐mediated regulation of iron homeostasis. Additionally, oxidative stress is known to disrupt iron homeostasis by suppressing the expression of bone morphogenetic protein 6 (Bmp6). Several studies have shown that oxidative stress exposure significantly decreases *Bmp6* mRNA levels, including in cultured retinal pigment epithelium (RPE) cells.^43^ Although this study's results indicate changes in *Bmp6* mRNA expression, the underlying molecular mechanisms remain unclear. This is because the study did not examine *Bmp6* expression at the protein level or functionally activate the BMP/SMAD pathway. The observed decrease in hepcidin expression alongside increased *Epo* expression is consistent with previous reports describing erythropoietic suppression of hepcidin. Mechanistically, *Bmp* ligands bind to type II *Bmp* receptors, which phosphorylate and activate type I receptors. Activated type I receptors phosphorylate SMAD1/5/8, allowing them to form complexes with SMAD4 and translocate to the nucleus to regulate transcription of BMP‐target genes, including hepcidin. Under conditions of increased erythropoiesis, hepcidin expression is suppressed. Reduced hepcidin stabilizes ferroportin (FPN1) on enterocytes, macrophages, and hepatocytes, thereby enhancing iron release into the circulation to support hemoglobin synthesis. Furthermore, hypoxia‐induced increases in *Epo* stimulate erythroid progenitor cells to produce and release erythroferrone (ERFE). ERFE neutralizes iron‐induced *Bmp6* activity, ensuring that hepcidin remains low and FPN1 stays stable. This coordinated mechanism ensures optimal iron availability for erythroblasts during erythrocyte production.^44^, ^45^, ^46^ This mechanism may be consistent with the findings of the present study; however, BMP6 protein levels, ERFE expression, SMAD phosphorylation, and downstream activation of the BMP/SMAD pathway were not evaluated. Therefore, the involvement of this pathway remains inferential and requires further mechanistic investigation. Despite these changes, a slight increase in Hemojuvelin *(Hjv)* gene expression was observed. This suggests that, in hemolytic anemia, although B*mp6* expression was suppressed, *Hjv* expression was relatively preserved (Figure 7). *Hjv* is an essential co‐receptor in the Bone Morphogenetic Protein (BMP) signaling pathway and plays a critical role in regulating hepcidin expression and maintaining iron homeostasis. Thus, the observed reduction in hepcidin expression may be more closely associated with decreased *Bmp6* expression than with alterations in *Hjv* expression. However, the functional activity of the BMP/SMAD pathway was not assessed in this study, and therefore, this interpretation should be considered with caution.^47^

In rats treated with sappanwood extract (SWE) at 50, 100, and 200 mg/kg BW, and with deferiprone (DFP) at 75 mg/kg BW, improvements were observed in several parameters. Administration of DFP at 75 mg/kg BW did not reduce hepatomegaly through the iron‐chelation pathway. Deferiprone is expected to chelate excess iron used in erythroid cell heme biosynthesis, which is subsequently incorporated into hemoglobin.^48^ The iron‐chelating and antioxidant effects of DFP in this study were practical, resulting in reduced hemosiderin aggregates and decreased total liver iron. Additionally, the antioxidant and iron‐chelating properties of DFP reduced direct, indirect, and total bilirubin levels by preventing hemolysis caused by free iron in serum. However, iron deficiency induced by deferiprone can disrupt hematopoiesis, including at the progenitor stage, by decreasing progenitor cell proliferation, clonogenic capacity, and survival.^49^ Therefore, DFP can suppress extramedullary hematopoiesis in rats. For SGPT and SGOT parameters, SGPT levels decreased by only 3% after DFP therapy. This may be due to deferiprone's ability to cross cell membranes and alter intracellular mitochondrial iron balance, potentially leading to mitochondrial dysfunction. This damage can trigger hepatocyte necrosis, leading to leakage of SGPT into the bloodstream. Thus, although DFP has protective effects against oxidative stress under specific conditions, it can exert the opposite effect by impairing mitochondrial function.^50^ In contrast, SGOT levels decreased by 11% with deferiprone therapy, a greater decrease than that observed with SGPT. This is likely due to additional physiological effects, such as reduced hemolysis, as reflected in SGOT levels, which are not liver‐specific.

Administration of SWE at doses of 50, 100, and 200 mg/kg BW exhibited variable effects on several observed parameters, including enzymatic activities and iron‐regulatory gene expression. The mechanism by which SWE reduces hepatomegaly involves iron chelation. In animal models of iron overload, administration of brazilin significantly decreased serum iron levels while increasing total iron‐binding capacity. Another contributing mechanism is brazilin's antioxidant activity. This compound has been shown to significantly reduce oxidative stress, as evidenced by decreased accumulation of reactive oxygen species (ROS) and lower malondialdehyde (MDA) levels, a marker of lipid peroxidation. Brazilin reduces H_2_O_2_ production by activating GPX7 (48), thereby lowering oxidative stress and preventing lipid peroxidation in erythrocyte membranes. Brazilin also increases erythrocyte deformability and ATP concentration.^50^ This mechanism may contribute to the maintenance of red blood cell membrane integrity and protection against free‐radical‐induced hemolysis. Previous studies have suggested that brazilin, a major bioactive compound in sappanwood, may interact with Janus kinase 2 (JAK2), a signaling molecule involved in the regulation of erythropoiesis and cellular survival. Molecular docking analyses have indicated that brazilin can bind to the ATP‐binding site within the catalytic domain of JAK2, suggesting a potential inhibitory interaction.^19^ However, the present study did not directly evaluate JAK2 activity or downstream signaling events. Therefore, any involvement of JAK2‐related pathways in the observed biological effects of SWE remains speculative and requires further experimental validation. Interestingly, although Bmp6 expression showed a non‐significant increase, Hamp expression was significantly elevated. This finding suggests that the regulation of Hamp expression may not be solely dependent on BMP/SMAD signaling. Additional regulatory pathways may also contribute to the observed changes in hepcidin‐related gene expression. However, the present study was not designed to identify the specific signaling mechanisms involved, and further mechanistic studies are needed to clarify the pathways underlying these effects. This finding suggests that other signaling pathways may contribute to hepcidin upregulation. According to Millot et al.,^51^ IL‐6 and endoplasmic reticulum stress also contribute to hepcidin induction via inflammatory pathways. Therefore, the increase in *Hamp* observed in this study may not be solely due to the BMP/SMAD pathway but may also be influenced by inflammatory responses. However, since this study focused on the *Epo* and BMP/SMAD pathways, these alternative mechanisms cannot be ruled out and require further investigation.

Interestingly, although all doses reduced iron levels, the 100 mg/kg BW group showed the most favorable overall response, whereas the 200 mg/kg BW dose did not yield further improvement and, in some parameters, exhibited a slight reduction in efficacy. This suggests a non‐linear dose–response relationship, in which increasing the dose does not necessarily result in a greater biological effect. A plausible explanation for this phenomenon involves pharmacokinetic and pharmacodynamic factors affecting the bioactive compounds in sappanwood extract. Brazilin, a moderately lipophilic compound (XlogP ≈ 1.50), may exhibit dose‐dependent differences in absorption, tissue distribution, and metabolic stability, potentially influencing its systemic availability. In addition, oral bioavailability may be limited by first‐pass metabolism occurring in both the intestinal wall and the liver. This process is mediated by metabolic enzymes, particularly cytochrome P450, which are expressed in both hepatic and intestinal tissues. Intestinal first‐pass metabolism may substantially contribute to overall metabolic extraction, thereby reducing the fraction of the active compound that reaches the systemic circulation and potentially limiting proportional increases in biological activity at higher doses.^52^

Furthermore, ferritin expression, including *Ftl*, is primarily regulated by the intracellular labile iron pool rather than total tissue iron content.^53^, ^54^ This explains why changes in *Ftl* expression, not directly parallel to total hepatic iron levels, may be observed in this study. The decrease in *Ftl* expression may therefore reflect reduced intracellular labile iron availability following iron chelation by sappanwood extract, rather than absolute changes in total iron stores. The relatively similar total liver iron levels observed between the 100 and 200 mg/kg BW groups further support this interpretation, indicating that total iron content does not necessarily reflect the metabolically active iron pool that regulates ferritin gene expression. This discrepancy suggests that sappanwood extract may preferentially affect labile iron dynamics, which are more relevant for cellular iron sensing and regulation. Overall, these pharmacokinetic limitations, together with potential saturation of absorption, first‐pass metabolism, and iron‐chelation dynamics, may contribute to the non‐linear dose–response relationship observed in this study, with 100 mg/kg BW as the optimal dose. However, these interpretations remain preliminary and require further pharmacokinetic and toxicological studies for confirmation.

Several limitations of the present study should be acknowledged. First, although the phenylhydrazine (PHZ)‐induced hemolytic anemia model reproduces clinically relevant features of hemolysis‐associated iron dysregulation, oxidative stress, and secondary hepatic iron accumulation,^55^ it does not fully recapitulate the complex pathophysiology of chronic iron overload disorders such as transfusion‐dependent thalassemia. In contrast to thalassemia, which is characterized by ineffective erythropoiesis, expanded erythroid activity, erythroferrone‐mediated hepcidin suppression, and progressive systemic iron overload, the PHZ model primarily induces acute oxidative hemolysis through direct erythrocyte damage. Therefore, the regulatory mechanisms of iron homeostasis in this model may differ from those observed in chronic disease states, particularly regarding long‐term erythropoietic signaling and hepcidin regulation. Accordingly, the present findings should be interpreted within the context of hemolysis‐induced iron dysregulation rather than as a complete representation of thalassemia pathophysiology. Second, the relatively small sample size and the use of a single animal model (male Wistar rats) may limit the statistical power and external validity of the findings. In addition, the absence of sex diversity further limits the generalizability of the results across biological populations. Third, the mechanistic interpretation of BMP/SMAD signaling is limited by the exclusive assessment of mRNA expression levels. Although changes in Hamp, Bmp6, and Smad4 transcripts suggest modulation of iron‐regulatory pathways, mRNA abundance does not necessarily reflect protein expression, post‐translational regulation, or functional pathway activation. Furthermore, circulating ERFE and serum hepcidin levels were not measured. As these biomarkers have been shown to correlate with iron status and hepcidin regulation in patients,^56^ the present findings are limited to hepatic gene expression and cannot directly confirm corresponding systemic hormonal changes. Therefore, the findings support transcriptional‐level changes but do not provide definitive evidence of downstream functional activation of the BMP/SMAD pathway. Furthermore, although hepatic iron content, Prussian blue‐based hemosiderin deposition, histopathological changes, and oxidative stress parameters were evaluated, pharmacokinetic properties, tissue distribution of bioactive compounds (including brazilin), and intracellular labile iron pools were not assessed. As a result, the mechanistic basis underlying the observed non‐linear dose–response relationship cannot be fully elucidated. The proposed explanations regarding bioavailability, first‐pass metabolism, and intracellular iron handling remain speculative, although they are consistent with the known pharmacokinetic behavior of polyphenolic compounds.

Finally, the relatively short duration of treatment limits assessment of long‐term efficacy, safety, and durability of the observed effects. Future studies incorporating larger cohorts, multiple disease models, pharmacokinetic and pharmacodynamic analyses, protein‐level validation of iron‐regulatory pathways, and extended intervention periods are warranted to strengthen mechanistic understanding and translational relevance.

## 5 CONCLUSIONS

Sappanwood extract shows hepatoprotective effects and helps regulate iron levels in phenylhydrazine‐induced hemolytic anemia. It reduces oxidative stress, improves liver function, and decreases hepatic iron accumulation. These effects are associated with the modulation of iron‐regulatory gene expression and the enhancement of antioxidant defenses. Overall, these findings suggest that sappanwood extract may be a promising natural iron‐chelating agent for managing iron overload and related hepatic complications.

## AUTHOR CONTRIBUTIONS

Mohammad Indra Pratama: Investigation, methodology, data curation, writing – original draft. Ratu Safitri: Conceptualization, study design, supervision. Desak Made Malini: Formal analysis, supervision. Mas Rizky A. A. Syamsunarno: Formal analysis, writing – review & editing. Mohammad Ghozali: Conceptualization, methodology, data curation, resources, writing – review & editing. Madihah: Data curation, formal analysis, project administration. All authors have read and approved the final manuscript.

## FUNDING INFORMATION

This research was funded by the Ministry of Higher Education, Science and Technology of the Republic of Indonesia through the BIMA Research Funding System, Grant No. 1640/UN6.3.1/PT.00/2025.

## CONFLICT OF INTEREST STATEMENT

The authors declare that there are no conflicts of interest regarding the publication of this paper.

## ETHICS STATEMENT

All experimental procedures involving animals were reviewed and approved by the Institutional Animal Care and Use Committee of Universitas Padjadjaran, under protocol number 414/UN6.KEP/EC/2025. All animal handling complied with the guidelines for the care and use of laboratory animals.

## GENERATIVE AI AND AI‐ASSISTED TECHNOLOGIES IN THE WRITING PROCESS

During the preparation of this manuscript, the authors used generative AI tools (ChatGPT and Grammarly®) to improve the clarity of language and grammar. After using these tools, the authors reviewed and edited the content as needed and take full responsibility for the publication's content.

## Data Availability

The data that support the findings of this study are available from the corresponding author upon reasonable request.
